# Supplementary figures and images for: Specification of distinct cell types in a sensory-adhesive organ important for metamorphosis in tunicate larvae
Source: PLoS Biol. 2024 Mar 13;22(3):e3002555. doi: 10.1371/journal.pbio.3002555 (PMC10962819; doi:10.1371/journal.pbio.3002555)

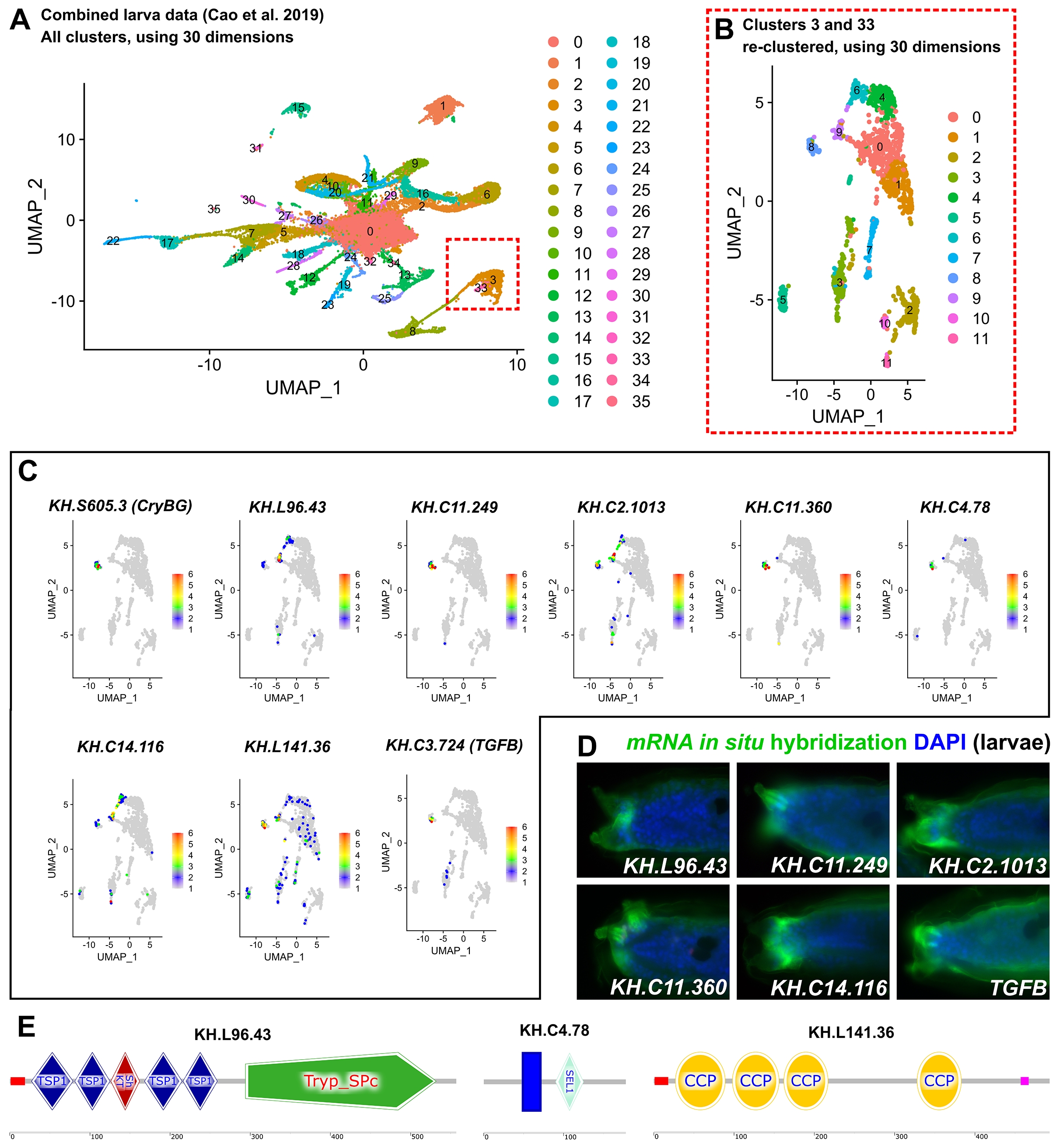

Supplement: S1 Fig — (A) Cell clusters based from reanalysis and re-clustering of whole-larva single-cell RNA sequencing (scRNAseq) data from Cao and colleagues (see S1 Data). Dashed red box indicated clusters 3 and 33, which appeared to correspond to several papilla cell types. (B) Cells from clusters 3 and 33 from plot A set aside and re-clustered. (C) Differential expression plots showing examples of candidate papilla cell type marker genes mapped onto clusters in B. (D) Fluorescent, whole-mount in situ mRNA hybridization (green) for certain genes plotted in C, labeling different cells in the papillae of Ciona robusta (intestinalis Type A) hatched larvae. (E) Protein domain prediction diagrams for select cell type-specific marker proteins generated by SMART [89]. Unless specifically named, genes are indicated by KyotoHoya (KH) ID numbers (e.g., KH.L96.43). All larvae were fixed at 18 h post-fertilization (hpf), 20 °C, except for C11.360 and C2.1013 (18.5 hpf). Blue counterstain is DAPI. (TIF) [file pbio.3002555.s001.tif]

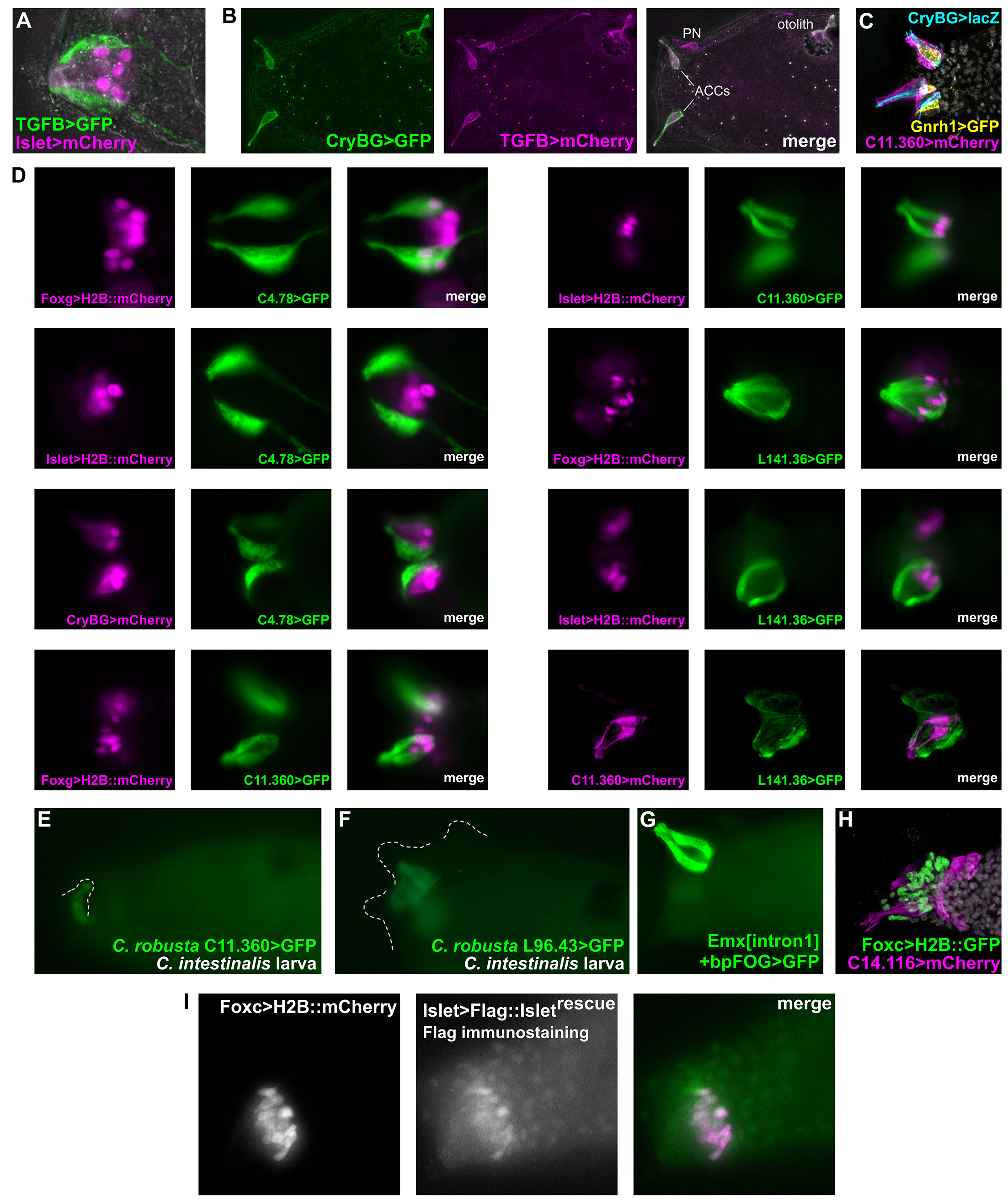

Supplement: S2 Fig — (A) TGFB>Unc-76::GFP reporter (green) is not co-expressed in the same cells as the Islet intron 1 + -473/-9>mCherry reporter (pink) at 20.5 hpf (~st. 29). (B) Cross-talk between CryBG>Unc-76::GFP and TGFB>Unc-76::mCherry reporter plasmids at 16 hpf (~st. 26), showing aberrant co-expression in ACCs and/or PNs only when co-electroporated. (C) Mutually exclusive expression of CryBG>lacZ in ACCs (cyan), Gnrh1>Unc-76::GFP in PNs (yellow), and C11.360>Unc-76::mCherry in ICs (magenta), with DAPI counterstained in gray. This larva is the same as in main Fig 2G, with an additional channel and different false coloring. (D) Images from Fig 2 with mCherry and GFP channels displayed separately. (E) C. intestinalis (Type B) larva electroporated with C. robusta C11.360>Unc-76::GFP reporter plasmid, showing specific but weak expression. (F) C. intestinalis (Type B) larva electroporated with C. robusta L96.43>Unc-76::GFP reporter plasmid, also showing weak expression. Papillae in panels E and F outlined by dashed lines. (G) Reporter plasmid containing the first intronic region of Emx drives expression in ICs at 20 hpf (~st. 29), likely corresponding to the “ring” of late Emx expression in Islet+ cells reported in Wagner and colleagues and distinct from earlier Emx expression in the papilla lineage as described in Liu and Satou. (H) C14.116>Unc-76::mCherry reporter expressed in central cells (ACCs+ICs, pink) and basal cells around the 3 papillae at 20.5 hpf (~st. 29). (I) Immunostaining for the Flag epitope tag fused to the Islet-rescue protein used for Islet>Islet experiments in Fig 4. Flag immunostaining in green and Foxc>H2B::mCherry in pink in merged image. Larvae fixed at 19 hpf (~st. 28). DAPI in gray. ACCs, axial columnar cells; PN, papilla neuron. (TIF) [file pbio.3002555.s002.tif]

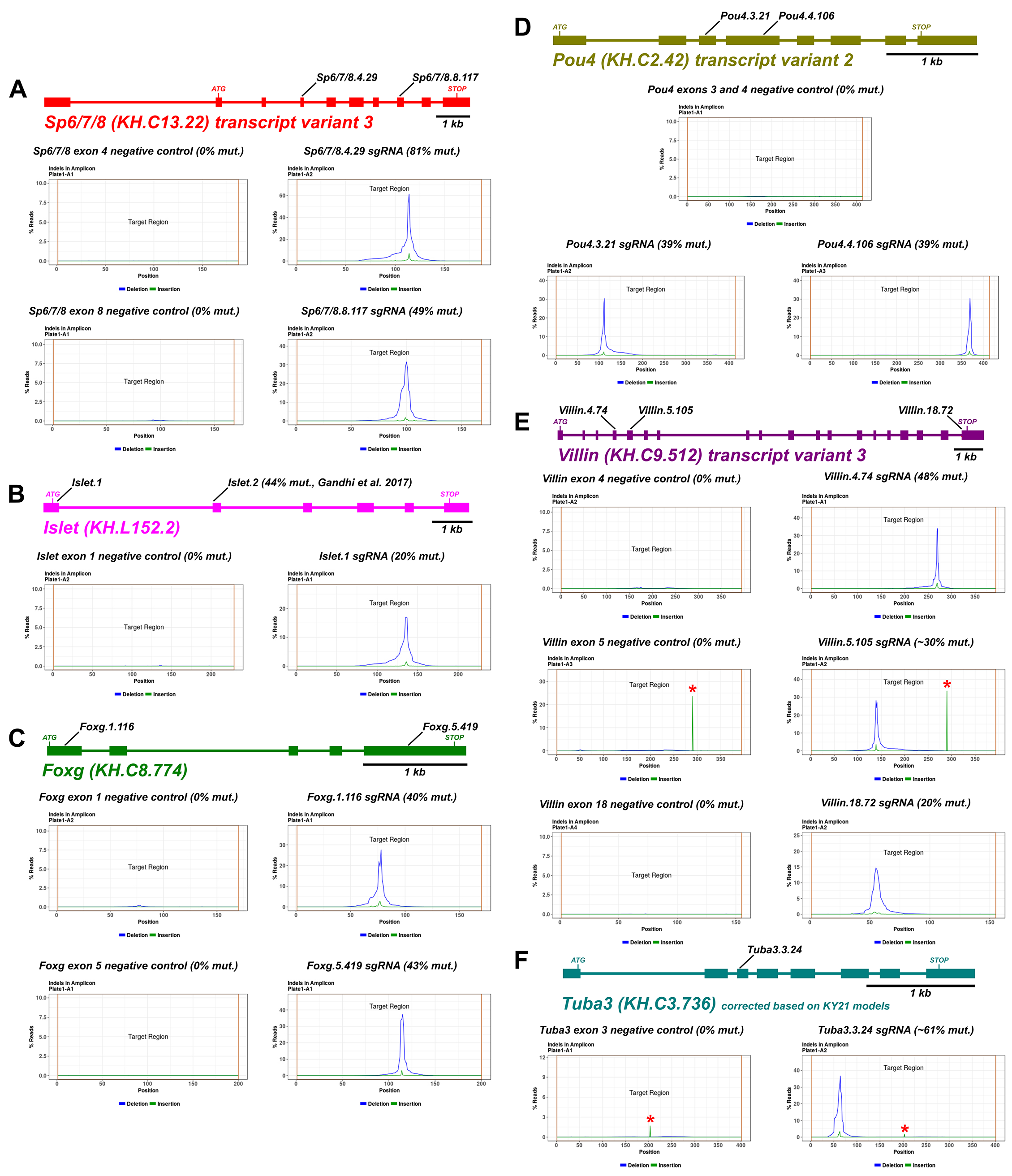

Supplement: S3 Fig — Gene loci diagrams for the 4 transcription factor-encoding genes investigated in this study: Sp6/7/8, Foxg, Islet, and Pou4. Plots underneath each gene show validation by Illumina sequencing (“Next-generation sequencing” or NGS) of amplicons, performed as “Amplicon-EZ” service by Azenta. Mutagenesis efficacies are calculated by this service, and histograms of mapped reads show specificity of indels elicited by each sgRNA. Negative control amplicons are amplified from samples that were electroporated with no sgRNA, U6>Control sgRNA, or sgRNAs targeting unrelated amplicon regions. Note different y axis scales for each plot. Asterisks in Villin exon 5 and Tuba3 amplicon plots indicate naturally occurring indels. Precise calculation of mutagenesis efficacy for Villin.5.105 and Tuba3.3.24 sgRNAs was not given due to these natural indels. (TIF) [file pbio.3002555.s003.tif]

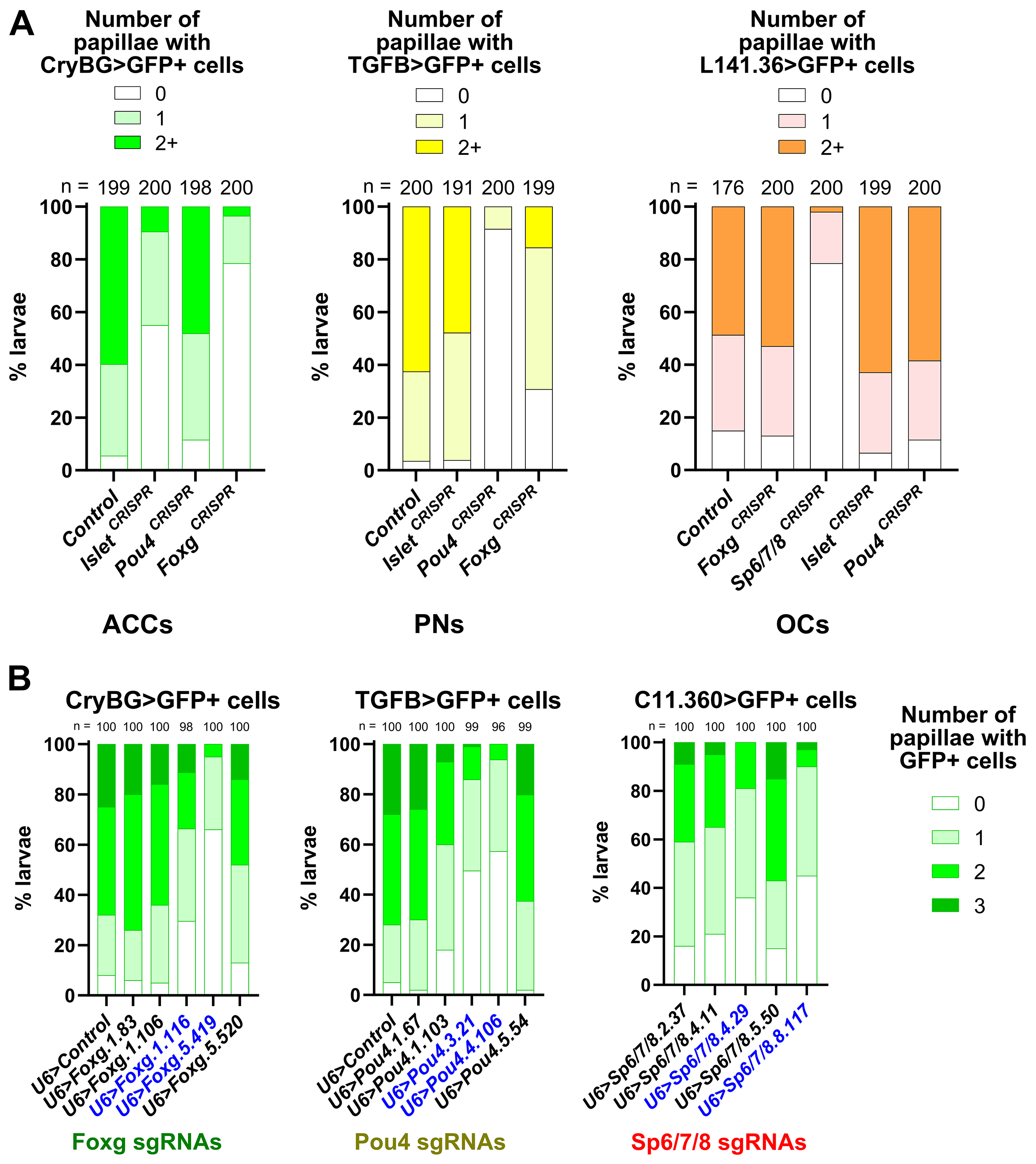

Supplement: S4 Fig — (A) Scoring of effect of papilla-specific CRISPR knockout of Foxg or Pou4 on specification of ACCs and PNs. Embryos were electroporated with Foxc>H2B::mCherry, Foxc>Cas9, CryBG>Unc-76::GFP (ACC reporter), TGFB>Unc-76::GFP (PN reporter), or L141.36>Unc-76::GFP (OC reporter), and gene-specific sgRNA combinations (see below for specific combinations). All were performed in duplicate and scores averaged, but some replicates and conditions are represented in Figs 3 and 4 also. Total embryos ranged between 76 and 100 per condition per replicate. Specific sgRNAs used: Foxg: U6>Foxg.1.116 + U6>Foxg.5.419; Pou4: U6>Pou4.3.21 + U6>Pou4.4.106; Sp6/7/8: U6>Sp6/7/8.4.29 + U6>Sp6/7/8.8.117; Islet: U6>Islet.2; Control: U6>Control. (B) Foxg, Pou4, and Sp6/7/8 sgRNAs were also tested alone (as opposed to pairs in combination) using reporter assays as in Figs 3 and 4. Those sgRNAs used further are highlighted in blue font. Additional sgRNAs abandoned due to low efficacy indicated in black font. For all plots, only larvae showing Foxc>H2B::mCherry expression in the papillae were scored. See S4 Data for the data underlying the graphs and for statistical test details. (TIF) [file pbio.3002555.s004.tif]

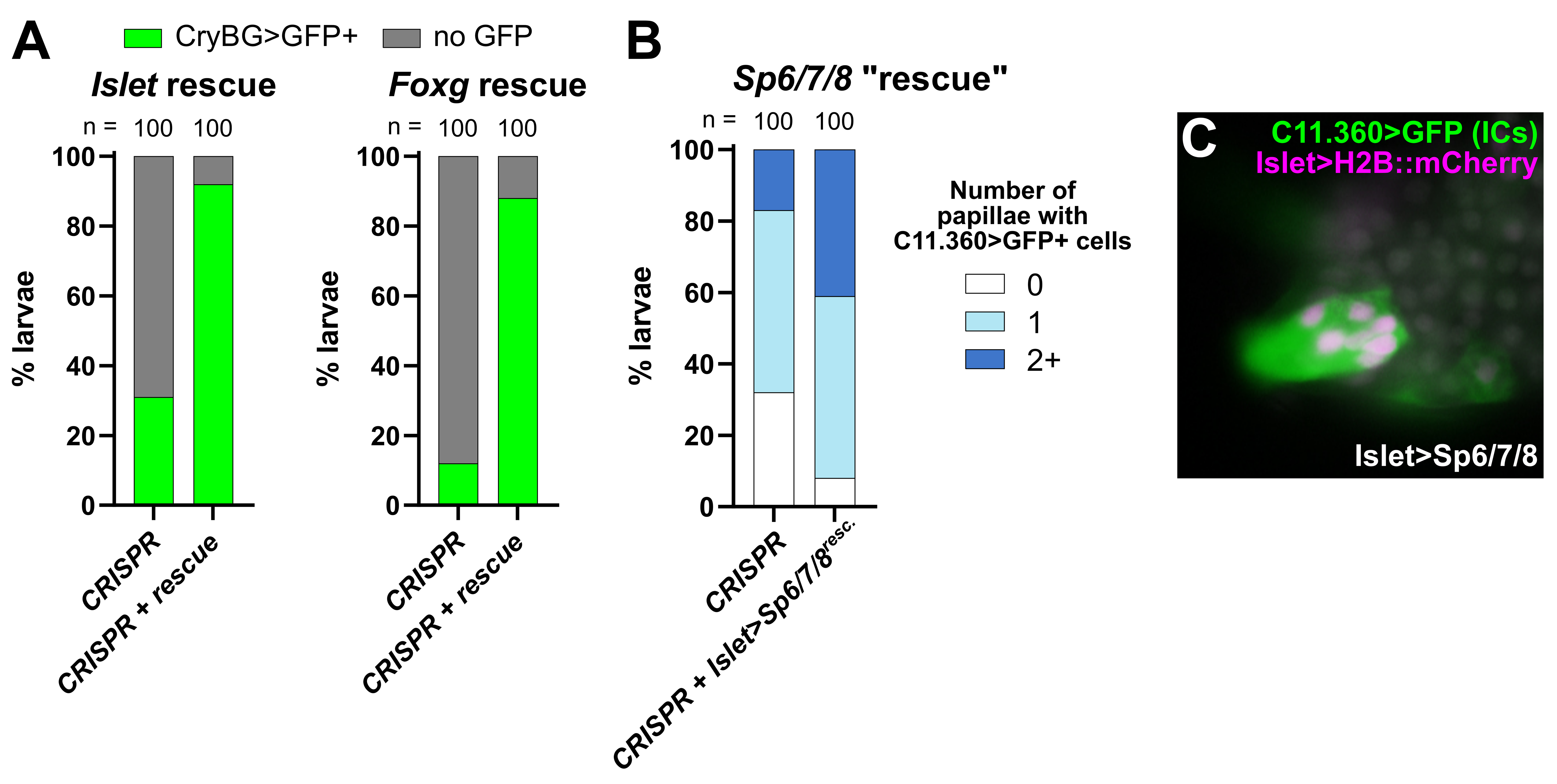

Supplement: S5 Fig — (A) CryBG>GFP expression in Islet (left) and Foxg (right) CRISPR larvae is rescued by co-electroporation with Islet intron 1 + bpFOG>Flag::Islet-rescue or Foxg>Foxg-rescue constructs, respectively, thanks to silent point mutations disrupting the sgRNA target binding sites. (B) Expression of C11.360>GFP is rescued in Sp6/7/8 CRISPR larvae upon co-electroporation with an Islet intron 1 + bpFOG>Sp6/7/8-rescue construct. (C) Example of expanded IC reporter (C11.360>Unc-76::GFP, green) in larvae (20 hpf/20 °C, ~st. 29) electroporated with Islet intron 1 + -473/-9>Sp6/7/8, as determined by perfect overlap with the Islet intron 1 + -473/-9>H2B::mCherry reporter (pink). See text for more details. See S1 File for exact sequences and detailed electroporation recipes. See S4 Data for the data underlying the graphs and for statistical test details. (TIF) [file pbio.3002555.s005.tif]

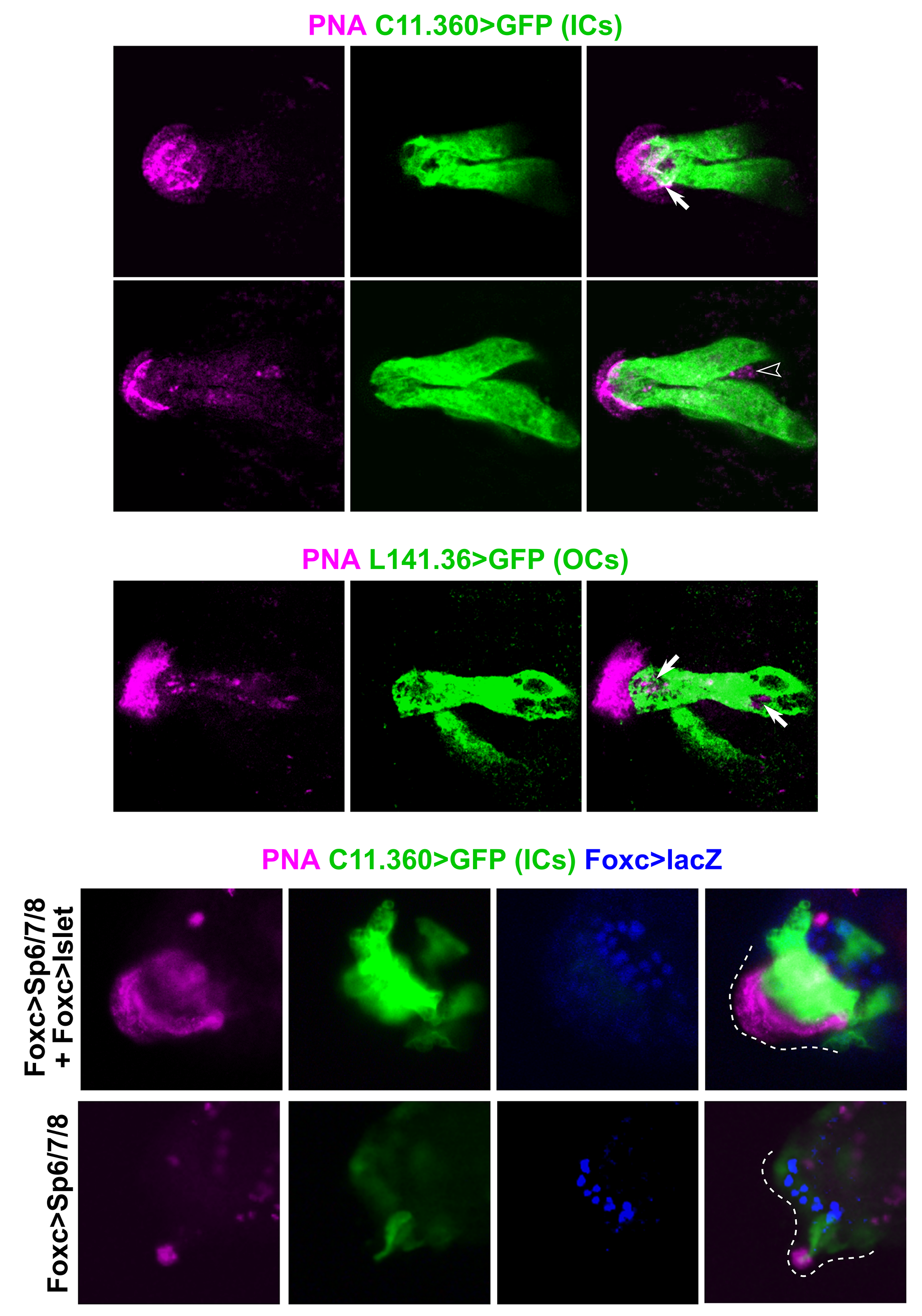

Supplement: S6 Fig — (TIF) [file pbio.3002555.s006.tif]

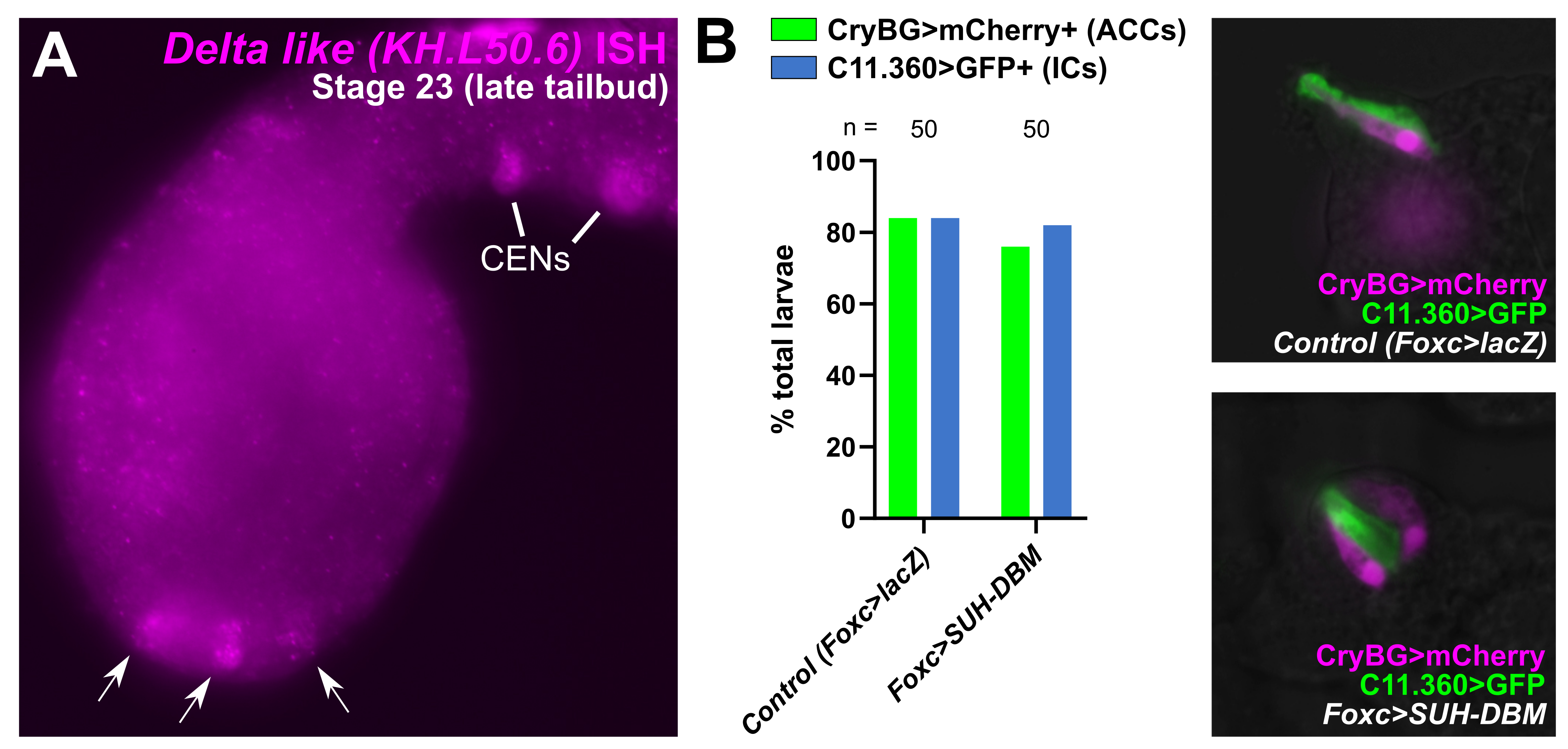

Supplement: S7 Fig — (A) Fluorescent whole-mount in situ mRNA hybridization for Delta like non-canonical Notch ligand (KH.L50.6) in a stage 23 embryo, marking epidermal sensory neurons including papilla neurons (arrows) and caudal epidermal neurons (CENs). (B) No significant difference in expression of either CryBG>mCherry or C11.360>GFP in larvae at 19 hpf at 20 °C (~st. 28) electroporated with the Delta-Notch pathway-inhibiting Foxc>SUH-DBM. Right: representative panels showing expression of both reporters in control and SUH-DBM-expressing larvae. See S4 Data for the data underlying the graphs and statistical test details. (TIF) [file pbio.3002555.s007.tif]

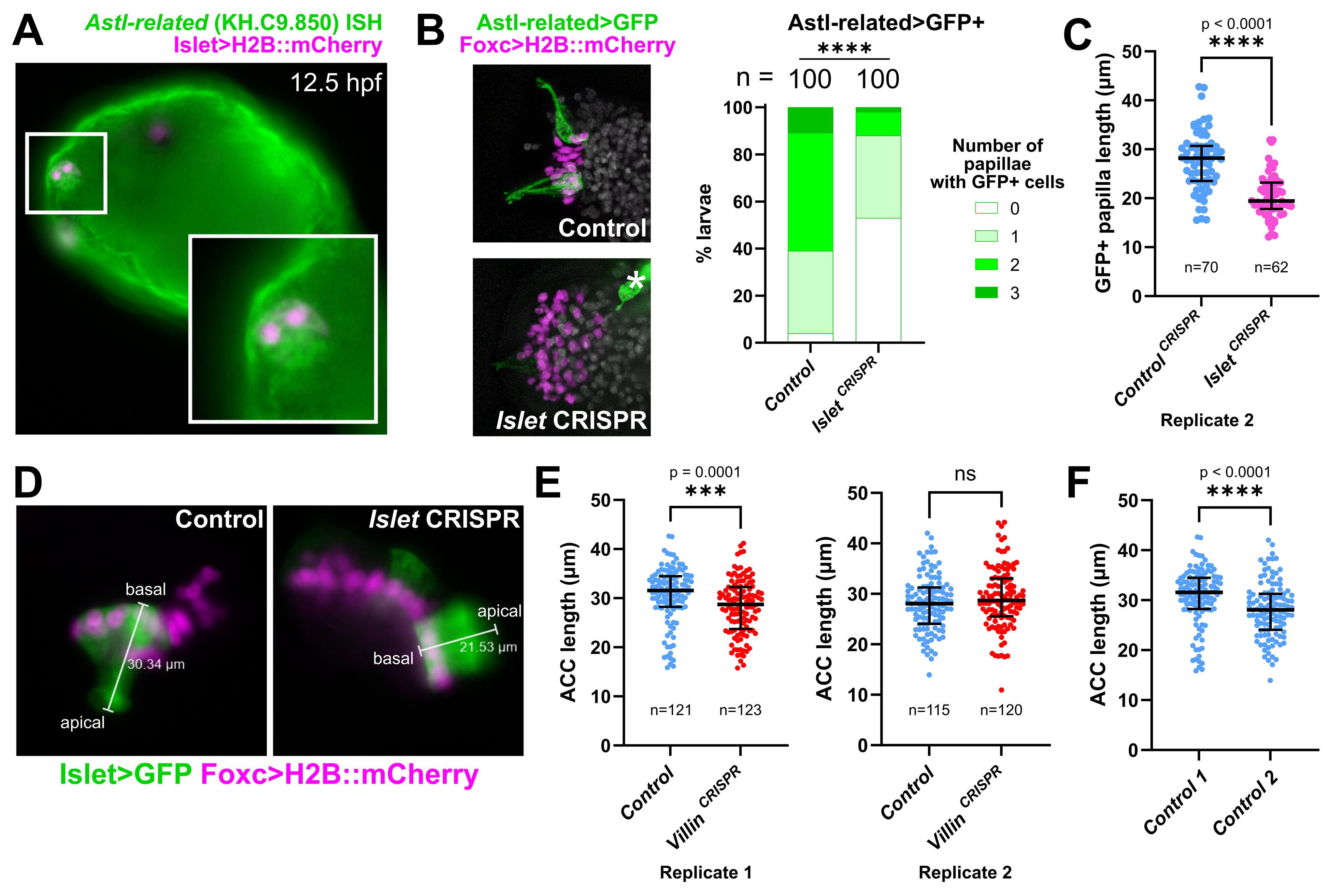

Supplement: S8 Fig — (A) In situ mRNA hybridization (ISH) showing expression of Astl-related (green) specifically in the Islet+ cells of the papillae (labeled by Islet intron 1 + -473/-9>mCherry, pink nuclei). (B) Tissue-specific CRISPR/Cas-mediated mutagenesis of Islet results in loss of Astl-related>Unc-76::GFP reporter expression in ACCs/ICs (green). Foxc>Cas9 was used to restrict CRISPR/Cas9 to the papilla territory (labeled by Foxc>H2B::mCherry, pink nuclei). Asterisk denotes residual reporter expression in cells outside the papilla territory. Right: Scoring of larvae represented in panel B, following criteria used for Fig 3. **** p < 0.0001 using chi-square test. (C) Second duplicate of Islet CRISPR experiment in Fig 7B. Statistical significance was determined by unpaired t test (two-tailed). (D) Representative images of control and Islet CRISPR larvae used for measurements in Fig 7B, with example of apical-basal cell length measurements. (E) Both duplicates of Villin CRISPR experiments side by side. Statistical significance was calculated using Mann–Whitney test (two-tailed) for replicate 1 and unpaired t test (two-tailed) for replicate 2. ns = not significant. (F) Comparison of ACC lengths measured in control larvae from the 2 duplicate Villin CRISPR experiments, showing statistically significant difference in average ACC lengths between different batches of larvae, calculated using Mann–Whitney test (two-tailed). See S3 and S4 Data for the data underlying the graphs and for statistical test details. (TIF) [file pbio.3002555.s008.tif]

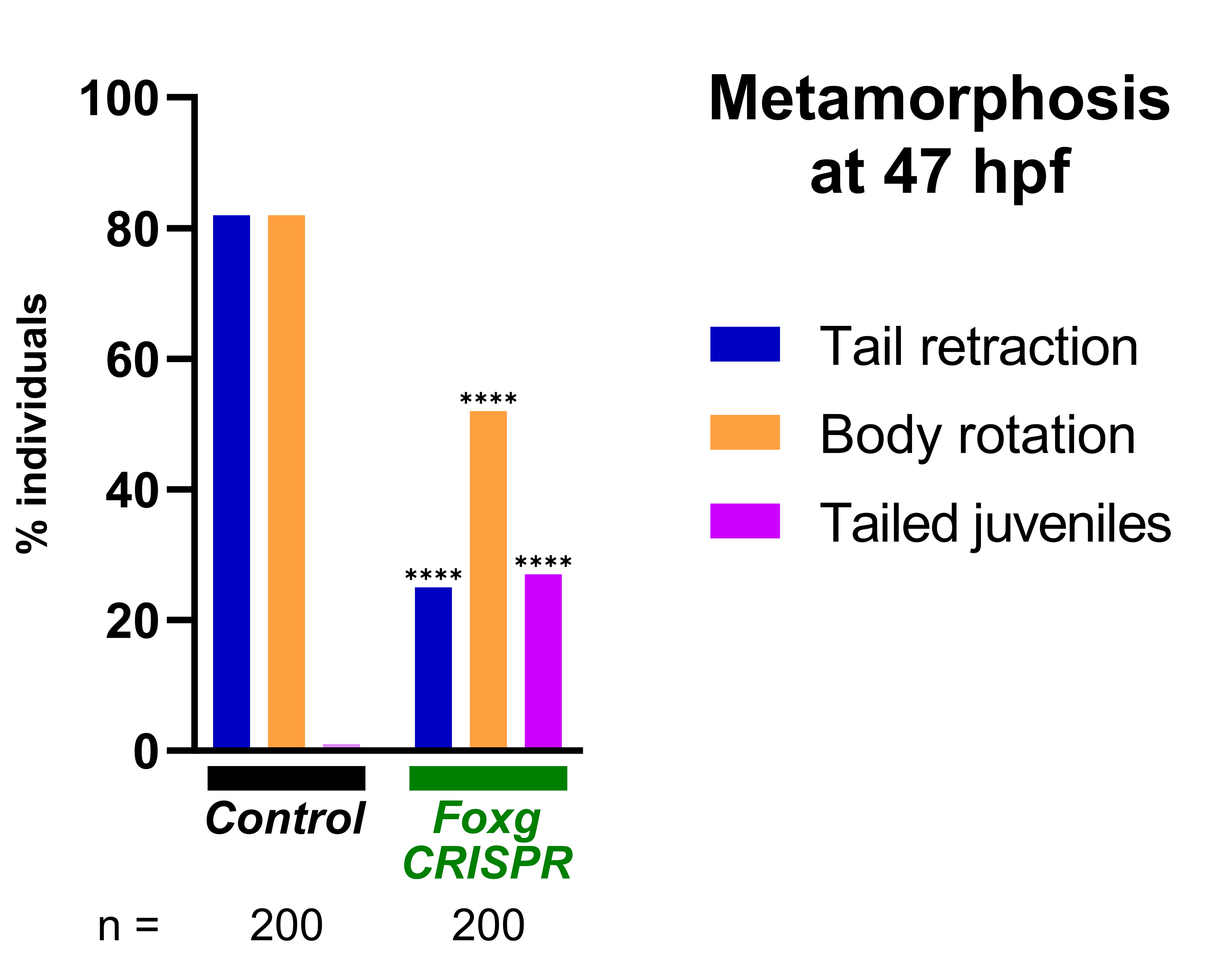

Supplement: S9 Fig — Scoring of Foxc>H2B::mCherry+ individuals as represented in Fig 7D, in third replicate of data in Fig 7B. See S1 File for detailed plasmid electroporation recipes. **** p < 0.0001 calculated by Fisher’s exact test. See S4 Data for the data underlying the graphs and for statistical test details. (TIF) [file pbio.3002555.s009.tif]

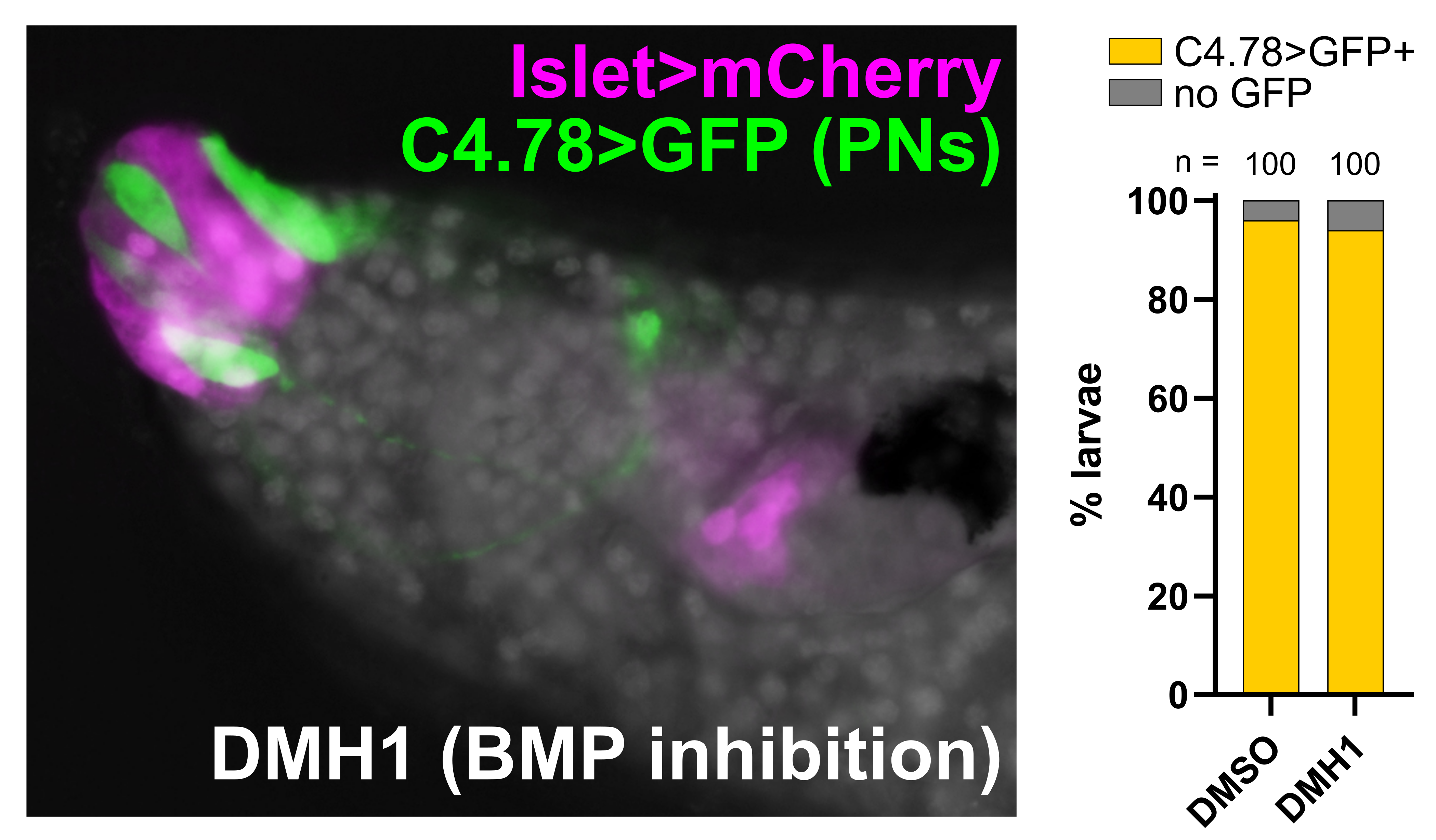

Supplement: S10 Fig — Larva co-electroporated with Islet intron 1 + -473/-9>mCherry (pink) and PN reporter C4.78>Unc-76::GFP (green) showing lack of substantial loss or expansion of PNs in larvae treated with the BMP pathway inhibitor DMH1, in spite of expanded Islet reporter expression and a single, enlarged papilla. Left: scoring of PN reporter expression in Islet>mCherry+ DMSO (negative control) and DMH1-treated larvae. All larvae raised to 19 hpf at 20 °C (~st. 28). See S4 Data for the data underlying the graphs and for statistical test details. (TIF) [file pbio.3002555.s010.tif]
