# Supplementary material for: Specification of distinct cell types in a sensory-adhesive organ important for metamorphosis in tunicate larvae
Source: PLoS Biol. 2024 Mar 13;22(3):e3002555. doi: 10.1371/journal.pbio.3002555 (PMC10962819; doi:10.1371/journal.pbio.3002555)
Supplement: Attachment — Submitted filename: ResponseToReviewers-Johnsonetal.docx [file pbio.3002555.s018.docx]

We thank the reviewers for their comments and suggestions. Please see below our point-by-point replies and explanation of changes.

***Response to Reviewer 1:***

***“Major comments:***

***1) Off-target effects of CRISPR/Cas9-mediated mutagenesis are not adequately addressed. The authors should consider validating each knockout/knockdown phenotype, for instance, by using another set of sgRNAs designed to target the same gene but differ in sequence from the ones used, or by performing genetic rescue experiments.”***

We have now updated the manuscript to address this. First, we performed genetic rescue experiments using mismatched cDNAs (cDNAs with silent point mutations disrupting the binding of relevant sgRNAs). In the case of *Islet* and *Foxg* CRISPR, we were able to use the genes’ own *cis­-*regulatory sequences to drive the rescue cDNAs’ expression, and indeed *Islet>Islet-rescue* and *Foxg>Foxg-rescue* both rescued loss of *CryBG>GFP* expression (new Supplemental Figure 5A). In the case of *Sp6/7/8,* we did not have a validated *Sp6/7/8* promoter, so instead we “rescued” the loss of *C11.360>GFP* (inner collocyte reporter) using an *Islet>Sp6/7/8-*rescue construct (new Supplemental Figure 5B). For *Pou4,* we also did not have the relevant *Pou4 cis*-regulatory element for a rescue experiment. However, we have included data showing that we get similar phenotypes with different individual sgRNAs targeting *Pou4* (in addition to similar data for *Foxg* and *Sp6/7/8,* see new Supplemental Figure 4B). Furthermore, a similar loss of PNs was previously reported for TALEN-based knock out of *Pou4* (Sakamoto et al. 2022)*,* therefore we hope these data are convincing in spite of lack of a true rescue.

***“2) Statistical significance of difference is not assessed in any of the experiments except for one (Fig. 7H). Thus, claims such as that expression was "substantially reduced relative to the control" (p.10 line 285) are not substantiated. In some cases, no quantification is provided at all. For example, the effects of U0126 on PN and OC marker expression, and expansion of the PN reporter expression by Foxc>SUH-DBM are not quantified or statistically assessed. Statistical assessment of significance is essential for evaluating the strength of support for each hypothesis being tested.”***

We have updated the manuscript to provide statistical support for all data. We have added all missing quantification, and all plots in main figures are averaged between duplicate experiments.

***“3) Evolutionary implications of the findings are unclear. The authors mention (in Discussion) hypotheses of homology between tunicate larval papillae and vertebrate structures (e.g. cement glands). However, it is unclear whether the findings of this study shed any insights relative to these hypotheses beyond providing resources or "a starting point for future investigations". Therefore, the findings of this study by themselves do not appear to have a broad impact beyond the field of ascidian development and neurobiology.”***

We have included a new paragraph in the discussion expanding these evolutionary questions, especially building upon the discussions from Liu and Satoh (2019) and Poncelet and Shimeld (2020). Perhaps our main novel finding is the common progenitor of papillae neurons and secretory OCs, which appears to be reflected in similarities between neural and secretory (exocrine) programs in vertebrates. However, as with any proposals trying to bridge long evolutionary distances, the evolutionary models are open to alternative interpretations. We have presented a nuanced discussion that is open to the possibility that the papillae are a tunicate-specific innovation, though we believe the regulatory networks themselves are more deeply conserved, likely descended from those involved in specification of ancestral neural/secretory cell types. One difficulty in confidently assigning homologies is the relative lack of detailed knowledge on gene networks specifying the various cell types in similar secretory ectodermal glands vertebrates (e.g. salivary, mucosal, lacrimal, mammary, sebaceous, cement, etc). However, this is why we believe our study could serve to ultimately provide strong support for such evolutionary connections proposed in the future.

***“4) Mutually exclusive expression and co-expression of genes are often unclear based on images presented in the figures. I suggest showing individual channels separately whenever demonstrating mutually exclusive expression patterns or co-expression patterns (e.g. Fig. 2A, D, G-J; Fig. 5A, B).”***

We have included new panels in Supplemental Figures 2 and 6 that show those figure with individual channels separated.

***“Minor comments:***

***Introduction:***

***- Page 3 line 71-72: "is" is missing between "which" and "required".”***

Fixed. Thank you.

***“Methods:***

***- Describe how each of the reporter constructs generated in this study was generated. Include information about the cis-regulatory sequences used.”***

Because a wide range of approaches were used to generate different constructs (e.g. restriction enzyme digest/ligation, homologous recombination, de novo synthesis, etc), we have instead provided the complete sequences of every sequence (coding and non-coding) used in the supplemental sequence file.

***“- Describe how many sgRNAs were used to induce knockout mutation for each target gene.”***

We have included this information in the methods, and detailed electroporation mix recipes are included in the supplemental sequence file as well.

***-“ Page 6: Describe what "KY" and "KH" are.”***

These just refer to two different versions/releases of the *Ciona robusta* genome assembly and gene models. We have tried to clarify this by describing them as KY21 and KH “versions” and giving their “full” names (“Kyoto 2021” and “KyotoHoya”, respectively).

***“Results:***

***- Page 8 line 218: What is the evidence that PNs are the only cell type bearing an axon in the papilla? Provide a reference.”***

We have now included the reference (Zeng et al. 2019b).

***“- Line 220: What is "cross-plasmid transvection"? Please explain.”***

This refers to the phenomenon of plasmid “cross-talk” that is sometimes observed in *Ciona,* but has never been formally described in publications, as far as we know. It is common knowledge that certain promoters on one plasmid will tend to activate transcription off a second, co-transfected plasmid. The term “transvection” is how this would be described if referring to endogenous loci in the genome, as it refers to interactions between allelic or nonallelic regions in different chromosomes, leading to transcriptional activation or repression. In our case, the plasmids are retained in the cells as extra-chromosomal arrays that might allow for such transvection to occur relatively frequently. We have solved this in the past by using certain basal promoters that seem refractory to such transvection (e.g. bpFOG). We have provided a brief explanation in the text, but as this phenomenon has not been published, we have no reference to cite.

***“- Line 222: What is the evidence that KH.C4.78 is a specific marker of PNs?”***

To clarify, the *KH.C4.78* reporter also labels the descending decussating neurons (ddNs) of the motor ganglion at the base of the tail, and also the bipolar tail neurons (BTNs). However, our images show that the reporter is always only expressed in PNs in the papillae, never in other papilla cell types. For this gene we did not attempt an *in situ* hybridization, but for the purposes of our studies, we were simply looking for a marker to label the PNs that did not label other peripheral neuron types like the RTENs (and that does not cross-talk with other reporter plasmids, like *TGFB*). We have clarified this by specifically using the term “reporter” instead of “marker” now.

***“- Page 9 line 245: Show evidence that L141.36 does not label PNs.”***

This is evidenced by the fact that this reporter never labels axons, whereas any other PN reporter or pan-papilla reporter (e.g. *Foxc>GFP*) does label axons. We have clarified this point in the text.

***“- Line 261: Which reporters label which cell types outside the papillae?”***

We have clarified that the PN reporter is expressed in other neurons in the tail and motor ganglion, while CryBG reporter is known to be expressed in the otolith.

***“- Page 10 line 302: Provide validation that Islet or Sp6/7/8 are indeed overexpressed.”***

Because it is true that the *Islet* overexpression results did not differ from the control for either ACC nor IC assays, the question arose of whether or not Islet was in fact being made. Therefore we repeated the experiments with a Flag-tagged Islet, and we show now by immunostaining that Islet protein is being made and is nuclearly localized as expected (Supplemental Figure 2G). We did not repeat for Sp6/7/8, however the strong, specific phenotype associated with this plasmid (loss of ACC reporter expression) does not raise much doubt that the plasmid is indeed working as designed.

***“- Line 304: Describe how expansion of IC reporter expression was quantified.”***

IC reporter expression area was determined to be larger than in the negative control, as judged by co-expression with the *Islet>H2B::mCherry* reporter. We show now a representative example in Supplemental Figure 5C.

***“- Page 11 Line 319: Show quantitative evidence that ACC reporter expression is normal in Sp6/7/8 mutants.”***

We apologize for the oversight. The data are now included in figure 4E.

***“- Line 322: Why should Foxc>Sp6/7/8 abolish IC reporter expression? Does it repress Islet (and Foxg)?”***

From Liu and Satou 2019, Sp6/7/8 does indeed repress *Foxg.* We believe that precocious overexpression of Sp6/7/8 may prevent *Foxg* from ever being activated, and this in turn would also prevent *Islet* activation and therefore loss of ACCs and ICs. However, it does not seem like Foxg is actually required for OC reporter expression (see *Foxg* CRISPR results in Supplemental Figure 4A). Hence we believe that this is why Sp6/7/8 overexpression does not abolish OC reporter expression.

***“- Line 324-325: Provide quantitative evidence that Foxc>Sp6/7/8 expands OC reporter expression.”***

Comparing the percentage of larvae showing “predominant” expression of the OC reporter between the negative control (Foxc>lacZ) and Foxc>Sp6/7/8, you can see a larger percentage (yellow bands at the top of the graph’s bars) in the latter. As we mention in the text, this was “slightly” expanded, and perhaps has more to do with the suppression of *Foxg* and the alternate fates that depend on Foxg (i.e. ACCs, ICs, and PNs to some extent, see Supplemental Figure 4A)

***- Page 12 line 353: Are ICs and OCs necessary for glue secretion? It seems possible to address this by perturbing the specification of both cell types using Sp6/7/8 CRISPR mutagenesis.”***

These are experiments that we are conducting for a follow up study focusing on the genetic basis of glue production.

***“- Line 367-368: This sentence is grammatically confusing as written. The three spots being referred to do not downregulate Foxg etc. I suggest changing it to "… arising from the cells that are in between… the three spots and downregulate Foxg…"”***

Fixed.

***“- Page 13 line 402: What about the effects of POU4 knockout on ICs and basal cells? Are there any?”***

We have not assayed these particular combinations. Here we prioritized investigating certain regulatory connections, but testing the effects of all knockouts on the inner collocytes will instead be prioritized in a future follow-up study centered on collocytes and glue production.

***“- Page 14 line 13: change in to is.”***

Fixed.

**“- 414: What is the expression pattern of Notch and Delta during papilla development?”**

Delta/Notch expression patterns assayed by *in situ* hybridization have been published as part of numerous studies including Imai et al. 2006 and 2009, Tang et al. 2013, etc, most of which can be found at the ANISEED database. We have included an *in situ* hybridization of our own, that of *Delta-like,* which encodes the major Delta ligand involved in lateral inhibition processes in the larval nervous system as shown in those other papers (Supplemental Figure 7).

***“- Line 417: Provide quantitative evidence that Foxc>SUH-DBM induces expansion of PN reporter expression.”***

We now include scoring data (in duplicate) in Figure 6K.

**“-Figure 7H -> 7F; Figure 7I -> 7G; Change Figure 7F -> 7H.”**

Since additional panels were added and re-lettered, we have changed most figure citations and carefully checked them all. We apologize for any errors or confusion.

***“- Line 474: Show evidence that Villin expression is reduced specifically in the central cells of the papillae upon Islet knockout.”***

We have now performed quantitative fluorescence intensity ratio measurements to show that *Villin* reporter is reduced in *Islet* CRISPR larvae (Figure 7F). Because the central cells were often the brightest in the control, the bulk of the fluorescence reduction would have occurred in the central cells. However, we could not identify the central cells when the reporter was lost from these cells, and their molecular identity was probably lost in *Islet* CRISPR anyway.

***“- Line 482: Provide representative images showing the difference in length along the apical-basal axis of ACCs between Villin CRISPR larvae and controls.”***

We have provided representative images of how we measured cell length (Supplemental Figure 8D) and all raw measurements in Supplemental Table 3. However, as you will see we have revised our interpretations based on repeating the *Villin* CRISPR and additional data on *Tuba3* CRISPR. We do not believe we have evidence, at present, to confirm the contributions of single effectors (like *Villin*) to papilla length, although we have new quantitative data showing a clear, reproducible effect in *Islet* CRISPR (Fig. 7B). We have retained these data in the revised manuscript in the spirit of transparency.

***“- Page 17 line 512: This implies that Sp6/7/8 CRISPR does not affect PN development, but was the effect of Sp6/7/8 CRISPR on PN development examined?”***

We include these data now in Figure 6I. *Sp6/7/8* CRISPR did not affect PNs as assayed by C4.78>GFP expression. We further discuss the implications in the Discussion.

***“Discussion:***

***- Page 20 line 602: How are PNs communicating with ACCs or ICs? Do PNs innervate ACCs or ICs - any relevant data (EM etc)?”***

Transmission electron microscopy in Zeng et al. 2019 showed PNs forming possible synapses onto the central cells. However, these local synapses have not been further investigated yet.

***“Figure 1:***

***- Indicate developmental stages in hours post fertilization as well, which would be helpful for readers unfamiliar with Ciona development to follow the manuscript.”***

We have done this throughout the manuscript, primarily in the figure legends. Because *Ciona* can be raised at any temperature ranging from 16 to 24 degrees, the exact correspondence between stages and hpf can vary. We have tried to report hpf, temperature, and stages, as reviewer 2 asked for stages, not hpf.

***“Figure 2:***

***- Line 666: Panel B does not show Foxg reporter expression.”***

We meant panel A. Fixed.

***“Figure 4:***

***- Line 703-705: Provide a quantitative definition for each category.”***

We have changed the “normal” category to “heterogeneous” and clarified that this corresponds to mixed on/off expression, under which all wildtype expression patterns are classified, but also reflects mixed on/off expression in situations that are not directly comparable to the wild type (e.g. when Islet overexpression results in a single, enlarged papilla).

***“Figure 5:***

***- I found it a bit odd that different species were used to examine PNA-stained granules in ICs (in C. intestinalis) and OCs (in C. robusta). Any reason?”***

The Rothbächer lab is in Europe, where *C. intestinalis* has been more easily obtained, while *C. robusta* is the predominant species available in the USA, where the Stolfi lab is located.

***“Figure 7:***

***- Line 769-770: The expression of Villin in Foxg+/Islet+ central papilla cells is not clear. Could Foxg and Islet expression be visualized together with Villin?”***

From looking at these papillae many times, it is our collective conclusion that these are central cells. Unfortunately we were not able to perform any double *in situ/*immunostaining for the revised manuscript.

***“Supplemental Figure 2:***

***- Line 1028: Label ACCs and PNs in the figure panels.”***

Fixed.

***“- Line 1035: What is the evidence that Emx reporter expression occurs in ICs?”***

Based on cell shape and position, the Emx>GFP+ cells did not resemble ACCs, PNs, nor OCs. Their morphology and position matched that of the ICs.

***Response to Reviewer 2:***

**“Major points:**

**- The Supplemental Sequence File is absent, preventing proper full evaluation.”**

Fixed. We apologize for the confusion.

***“- The number of independent experiments that have been performed is not indicated. From Fig S6, it seems replicates have not been generally done. This is a serious issue that needs clarification from the authors.”***

To clarify, Figure S6 was actually a third replicate, though data from the two replicates were pooled previously. However, we have now performed all experiments in the main figures in duplicate and present these as averages of percentages. Statistical significance tests are presented in the methods and figures. Repeating experiments in duplicate did not alter the majority of conclusions with two notable exceptions:

1) Repeating the experiment with a third color reporter (Beta-gal immunostaining) and re-imaging using a better microscope after the original microscope turned out to be defective and no longer functioning resulted in a more conclusive finding that eliminating ICs dampens PNA staining, while converting the entire papilla results in strong ectopic PNA staining throughout. With this, we conclude that the ICs are a major contributor to the PNA-stained adhesive material of the papillae.

2) Repeating the cell measurements in *Villin* CRISPR showed a non-significant difference between CRISPR and control, but it turns out that this was entirely due to a significant difference in the controls between the two batches. The CRISPR cell lengths were remarkably consistent. We do not believe we are missing some kind of unconscious bias- larvae were fixed at the same stage and cells were imaged randomly. Furthermore, measurements repeated on old images resulted in the same difference, indicating it is not a measurement problem. We believe that the ACCs dynamically contract in length, and evidence from our own work (Johnson et al. 2020) and others (Hoyer et al. 2023) suggests the ACCs may be responding to external cues. It is possible that in one batch, control ACCs were sensing different cues (perhaps the larvae were more crowded, or there was more bacteria in the water) and that in one batch those ACCs responded differently.

Because of this, we further tested another candidate effector of cell length, the alpha Tubulin gene *Tuba3,* and found no statistical difference between CRISPR and control. Even though our new measurements on *Islet* CRISPR larvae show stark cell length differences. We think that these single effector gene perturbations might not result in any changes in cell length. This is because the genes are not involved in regulating cell length, and/or because a combination of Islet targets regulate cell length in a redundant manner that is robust to individual gene knockouts. Although these results are inconclusive, we have kept them here for the sake of transparent reporting.

***“- Given the efficient perturbation (CRISPR and overexpression) and analysis tools that have been produced, it is a pity that the fate of all cell types has not been systematically examined. While I understand that basal cell formation could deserve a specific study, additional data would make the study stronger. More specifically: - Fig 4: PNs are missing in the analysis.”***

Indeed the basal cells are outside the scope of this study as they do not arise from *Foxg+* progenitors and therefore are usually considered outside the papillae proper. For the PNs, we have now included data on *Sp6/7/8* CRISPR effect on KH.C4.78>GFP expression (PN reporter), and it appears that Sp6/7/8 is not required for PN specification (Figure 6I). The implications are further discussed in the Discussion. The only other cell type for which we do not have complete CRISPR data are the ICs. These are the focus for a follow up study specifically on glue formation and collocytes.

***“- Fig 6: the role of Notch signaling pathway in the formation of ACCs and ICs has been eluded.”***

We had previously performed preliminary Notch perturbation experiments looking at ACCs/ICs and did not notice any substantial effect. We have now included these experiments in Supplemental Figure 7. Foxc>SUH-DBM did not appear to have any noticeable effect on ACC/IC reporter expression. We still do not have a good lead on signaling pathways involved in ACC/IC fate choice. Preliminary experiments, even when interpretable, have not yielded any firm conclusions. This cell fate decision will need to be followed up with a more careful, thorough set of experiments.

***“- The role of Sp6/7/8 using CRISPR deserves more detailed attention. According to Liu and Satou (2019), Foxg and Isl should be ectopically expressed (the U-shaped swath). Is it the case using CRISPR? In that case, there should be no PNs (not examined, same point as above), and additional ACCs (but it is not the case in Fig 4). Without PNs the metamorphosis should be inhibited, but it is pretty normal (Fig 8). Since Sp6/7/8 has a dynamic expression pattern, a possible explanation is that CRISPR experiments only display phenotypes in the collocytes, the latter/massive domain of expression of Sp6/7/8. This aspect should be explained/discussed by the authors.”***

Indeed, we discuss this in the Discussion section. Based on our interpretations of Liu and Satou 2019 and our own new data on lack of *Sp6/7/8* CRISPR effect on PN reporter expression (Figure 6I), we think that even though Sp6/7/8 represses *Foxg,* FGF/MAPK likely represses *Islet* in parallel. Thus *Sp6/7/8* CRISPR would not be sufficient to expand *Islet* the same way that U0126 does. The regulatory connections between *Sp6/7/8, Foxg, Islet,* and the neurogenic program deserve their own careful follow up study.

***“- The authors have assigned cell lineage to the different cell types through indirect evidence mainly based on gene expression patterns/gene function. This is quite reasonable, and I am not asking for direct lineage tracing experiments. However, the case of PNs is questionable. The lack of PNs following MAPK inhibition leads the authors to trace PNs to the missing territory (Foxg+ then Foxg- cells). This is in contradiction with a recent report (Roure et al., 2023) that shows a similar Foxg+/Isl+ U-shaped stripe surrounded by potential Pou4+ PNs following BMP inhibition. The authors propose that these Pou4+ cells are not PNs but other neurons (RTENs). It is also very possible that MAPK inhibition blocks PNs specification/formation. The authors have potentially three ways (MAPK inhibition, BMP inhibition and Sp6/7/8 CRISPR) to produce a Foxg+/Isl+ U-shaped stripe. It would be crucial to determine in such embryos the expression of Pou4 and PN specific markers (KH.C4.78 for example).”***

We have now repeated the BMP inhibition from Roure et al. (DMH1 inhibitor treatment), looking at our PN marker *KH.C4.78>GFP* (Supplemental Figure 10)*.* In this case, we did not observe either expansion of PNs nor loss of PNs, even though *Islet* was expanded to form a single enlarged papilla. Based on these data, our interpretation is that DMH1 and U0126 treatments result in slightly different effects. We propose that the expansion of *Pou4* seen in Roure et al. may reflect RTEN-like cells or undifferentiated neural progenitors, not fully differentiated PNs. Unfortunately we do not have a *Pou4* reporter and we were not able to perform *Pou4 in situs* to carefully test this*.* Roure et al. also discuss the idea that BMP may act primarily on the ventral papilla, and indeed we did not see any clear indication of PNs in the ventral areas in our DMH1-treated larvae; *KH.C4.78>GFP* expression seemed to always occur around the dorsal papilla areas, though we were not able to quantify this yet. We still believe that PNs arise from Foxg-downregulating cells due to: 1) The position of *Pou4+* cells in wildtype larvae in between *Foxg+* central cells (though the dorsalmost PNs might be an exception, as they may arise from cells that are dorsal to the dorsal-most *Foxg+/Islet+* spots); 2) The concordance between loss of OCs and gain of PNs in *Foxc>SUH-DBM* (now quantified better in Figure 6J,K); 3) The similar frequency of loss of OCs and PNs in U0126-treated larvae; and 4) Consistent expression overlap of PN reporters with *Foxg* reporters but not *Islet* reporters.

***“Minor points:***

***- Staging is overall poorly described, mainly by indicating XX hpf at XX°C. It is necessary to indicate stages, possibly by using the staging system defined in Hotta et al. 2007.”***

We have updated the figure legends to describe both staging and hpf/temperature for all figures. Reviewer 1 raised the point that those unfamiliar with Ciona prefer hpf. Unfortunately the late larval stages described in TUNICANATO (Hotta et al. 2020) were based on incubation at 18 degrees, not 20 as we do with *C. robusta* in our lab. Therefore we have estimated the approximate Hotta/TUNICANATO stages for all larvae and juveniles based on what we believe are the correct correspondences between 18 and 20 degrees.

***“- Line 182: mounting method is not described earlier in the Methods section.”***

This has been fixed.

***“- Lines 207-208: it would be interesting to describe with more details the novel cell type markers (protein domains, phylogenetic distribution (Ciona specific? ascidian specific?...).”***

We have included some information about each marker gene, and diagrams of protein domain predictions in Supplemental Figure 1E, with the exception of KH.C11.360 which does not contain any conserved protein domains. Although none of these markers have clear homologs in mammals, we can say that some appear conserved outside tunicates. For instance, KH.C4.78 is found in non-mammalian vertebrates. However, a careful phylogenetic analysis would be required to assign clear orthologies and understand which phyla have or do not have them, especially as some of these markers have “modular” domains that are frequently found in various different proteins.

***“- C. robusta vs C. intestinalis cis-regulatory sequences. Without the supplemental sequence file, it is difficult to appreciate the type of regions used (position in the locus, size...). Could the authors show comparisons of the respective loci with an evaluation of their conservation at the nucleotide level?”***

Again we apologize for not initially uploading the supplemental sequence file. We have included the *cis*-regulatory sequences from both species, and in the text we explain the % identity between these regions disregarding any large species-specific gaps or insertions. For KH.L141.36, it is ~66%, for KH.C11.360 it is ~89%.

***“- Fig 4 and line 319: could the authors show a quantification of the effect of Sp6/7/8 CRISPR on ACCs as in Fig 4E?”***

Yes, we apologize for the omission. We have included now scoring as for the other conditions/reporters.

***“- It seems that analyses of the phenotypes have been generally performed on embryos/larvae that were positive for an 'electroporation tracer' such as Foxc>H2B::mCherry. The authors should check that is explicit in all figure legends. It seems missing in Fig 4 for example.”***

Yes, indeed with rare exceptions (e.g. Supplemental Figure 7B), scoring was always performed on mCherry+ individuals (or B-gal+, in the case of PNA experiments) as the “tracer”. We have made this more explicit in the methods section, and have clarified this in all the figure legends.

***“- Could the authors comment on the number and diversity of the cell types that they have uncovered? They have clearly identified 5 cell types from a sc-RNAseq re-analysis that yields 11 clusters (Fig S1). Are there additional cell types? Any hint of differences for a given cell type, for example along the dorso-ventral or left/right axes?”***

To clarify, Fig S1B contains much more than just papilla cell types. The vast majority of the cells in this sub-analysis is from outside the papillae. Judging from the plots of gene expression in Fig S1C, we believe the whole papilla territory (including basal cells) only encompasses subclusters 6, 8, and 9 in S1B. We did not find any evidence for inter-papilla differences, especially as many of the “single cells” appear to be doublets/triplets (possibly due to the tight junctions between the papilla cells, or adhesion by the glue) and resolving the expression domains of the different markers required *in situs* and reporters.

***“- The model presented for the regulation of ACCs, ICs and OCs by Islet and Sp6/7/8 is very nice. Could the authors comment on results that do not have a perfect match. An increase of ACCs (at the expense of ICs) is expected in Sp6/7/8 CRISPR, but it isn't the case. Similarly, an increase of OCs (at the expense of ICs) is expected in Isl CRISPR, and it does not occur.”***

Indeed, we discussed this above and in the Discussion- the idea that Sp6/7/8 might not actually be required to repress *Islet,* and that restriction of *Islet* expression may be carried out by FGF/MAPK independently of Sp6/7/8. Similarly, we do not believe Islet is required for restricting OC/PN fate.

***“- Possibly again because of the absence of the supplemental sequence file, it is unclear which sgRNA (alone or in combination) has been used for each result that is presented. Also, when using a different sgRNA (or a combination instead of a single one) to target the same gene, the difference in phenotype penetrance/strength is not indicated. If not tested, it should be clearly stated (for example line 447).”***

We have now clarified this in the Methods section, and noted exceptions in relevant figure legends. In the majority of cases, all sgRNAs validated in Supplemental Figure 3 were used in combination. For *Islet* CRISPR*,* only the *Islet.2* sgRNA was used for most of the experiments as the *Islet.1* sgRNA was only validated later. The combination *Islet.1* + *Islet.2* was used only for new data added in the revision, namely quantification of papilla cell length in *Islet* CRISPR (Figure 7B, replicate in Supplemental Figure 8), and quantification of *Islet* CRISPR effect on *Villin* reporter expression (Figure 7F).

***“- Fig 6H: asterisk not described in the legend.”***

We have fixed this. It just shows leaky/background GFP expression in mesenchyme cells near the papillae.

***“- Cell elongation (Fig 7). The authors focus on ACCs/ICs. But, it seems clear from their data that OCs and PNs are also elongated. Furthermore, basal cells are also more elongated than 'typical epidermis' outside the papilla region. In the Isl CRISPR, cells seem similar to basal cells. This suggests, that there may be a least two levels of cell shape control, one general to the papilla organ and one specific to the 3 protrusions.”***

Indeed, we have added this observation (based on Wagner et al. 2014) in the text. Many of these targets we see (*Villin, Tuba3,* and others) seem to be expressed broadly earlier, possibly activated by Foxc initially, then Foxg. Untangling these factors and their control of papilla morphogenesis will require a detailed follow-up.

***“- Line 443: Fig 7C does not show the RNAseq design.”***

Fixed.

***“- Line 449: 'Islet conditions', what does it mean?”***

We have removed this, as it alludes to additional RNAseq conditions that were not included in this study.

***“- Line 455: references of already validated ACC genes seem missing. These genes could be highlighted in Supplemental Table 2.”***

We have now highlighted previously validated ACC genes in green, and newly identified markers in orange.

***“- Lines 471-472: wrong figure panels.”***

This part has been revised with new, more quantitative data and the figure panel referencing fixed.

***“- Are Astl-related and Villin expressed in both ACCs and ICs?”***

By *in situ,* their expression occurs in the common progenitors of ACCs/ICs.

***“- ACC length measurement is not fully convincing since it seems to depend largely on the orientation of the larva and the ACCs during imaging. Also the number of cells that have been measured is not indicated. The numbers of papillae, larvae and experiments need also to be described.”***

We have updated the Methods to include detailed descriptions and approach used. Briefly, ACC clusters were measured individually. A larva could have up to 3 GFP+ papillae, and as such three measurements would be gathered from such a larva, but we could not resolve individual cells with this approach. Indeed the measurements varied from papilla to papilla, but these differences are averaged out in our large enough “n”, and our data show remarkable consistency with the exception of one control batch which we explain above. In the end, we believe ACC contraction in response to external stimuli may be a variable that is difficult to control for.
